# 3-(4-Chloro­phen­yl)-7-methyl-4-(4-methyl­phen­yl)-1-oxa-2,7-diaza­spiro­[4.5]dec-2-en-10-one

**DOI:** 10.1107/S1600536807068717

**Published:** 2008-01-09

**Authors:** D. Gayathri, D. Velmurugan, R. Ranjith Kumar, S. Perumal, K. Ravikumar

**Affiliations:** a Centre of Advanced Study in Crystallography and Biophysics, University of Madras, Guindy Campus, Chennai 600 025, India; b Department of Organic Chemistry, School of Chemistry, Madurai Kamaraj University, Madurai 625 021, India; c Laboratory of X-ray Crystallography, Indian Institute of Chemical Technology, Hyderabad 500 007, India

## Abstract

In the title compound, C_21_H_21_ClN_2_O_2_, the dihydro­isoxazole ring adopts an envelope conformation and the piperidinone ring is in a chair conformation. The dihedral angle between the two benzene rings is 84.2 (1)°. The crystal used was an inversion twin.

## Related literature

For general background, see: Diana *et al.* (1985 ▶); Huisgen (1984 ▶); Lepage *et al.* (1992 ▶); Ryng *et al.* (1998 ▶); Torssell (1988 ▶). For puckering parameters, see: Cremer & Pople (1975 ▶). For asymmetry parameters, see: Nardelli (1983 ▶).
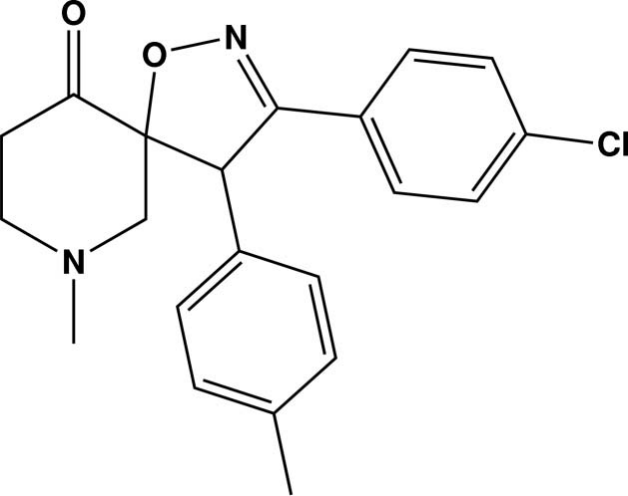


## Experimental

### 

#### Crystal data

C_21_H_21_ClN_2_O_2_
*M* *_r_* = 368.85
Orthorhombic, 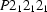

*a* = 11.4585 (8) Å
*b* = 16.1132 (11) Å
*c* = 10.1038 (7) Å
*V* = 1865.5 (2) Å^3^
*Z* = 4
Mo *K*α radiation
μ = 0.22 mm^−1^
*T* = 293 (2) K
0.24 × 0.23 × 0.20 mm

#### Data collection

Bruker SMART APEX CCD area-detector diffractometer
Absorption correction: none
16236 measured reflections
4350 independent reflections
3771 reflections with *I* > 2σ(*I*)
*R*_int_ = 0.022

#### Refinement

*R*[*F* ^2^ > 2σ(*F* ^2^)] = 0.043
*wR*(*F* ^2^) = 0.111
*S* = 1.03
4350 reflections
238 parameters
H-atom parameters constrained
Δρ_max_ = 0.24 e Å^−3^
Δρ_min_ = −0.16 e Å^−3^
Absolute structure: Flack (1983 ▶), with 1846 Friedel pairs
Flack parameter: 0.65 (6)

### 

Data collection: *SMART* (Bruker, 2001 ▶); cell refinement: *SAINT* (Bruker, 2001 ▶); data reduction: *SAINT*; program(s) used to solve structure: *SHELXS97* (Sheldrick, 2008 ▶); program(s) used to refine structure: *SHELXL97* (Sheldrick, 2008 ▶); molecular graphics: *PLATON* (Spek, 2003 ▶); software used to prepare material for publication: *SHELXL97* and *PARST* (Nardelli, 1995 ▶).

## Supplementary Material

Crystal structure: contains datablocks I, global. DOI: 10.1107/S1600536807068717/ci2543sup1.cif

Structure factors: contains datablocks I. DOI: 10.1107/S1600536807068717/ci2543Isup2.hkl

Additional supplementary materials:  crystallographic information; 3D view; checkCIF report

## Figures and Tables

**Fig. 1.**
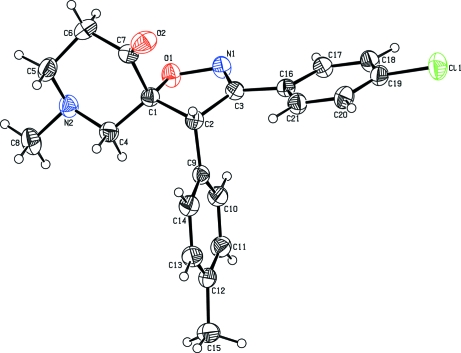
The molecular structure of the title compound, showing 30% probability displacement ellipsoids.

**Fig. 2.**
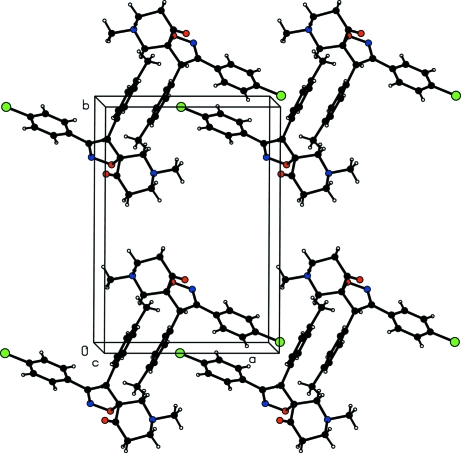
The crystal packing of the title compound viewed down the c axis.

## supplementary crystallographic information

### Comment

1,3-Dipolar cycloaddition of nitrile oxides to alkenes and alkynes affords isoxazoles and isoxazolines (Torssell, 1988). Apart from exhibiting important biological activities such as antiviral (Diana *et al.*, 1985), anticonvulsant (Lepage *et al.*, 1992) and immunostimulatory (Ryng *et al.*, 1998), isoxazolines are valuable synthons in the synthesis of α,β-unsaturated ketones, β-hydroxy ketones and γ-amino alcohols (Huisgen, 1984). In view of the above facts, we have undertaken the X-ray crystal structure determination of the title compound.

The sum of the bond angles around N2 (331.7°) indicates the *sp*^3^-hybridization. The dihydro-isoxazole ring (C1—C3/O1/N1) adopts an envelope conformation with atom C1 deviating by 0.350 (2) Å from the plane of rest of the atoms in the ring. The piperidinone ring adopts a chair conformation. The puckering parameters (Cremer & Pople, 1975) and the smallest displacement asymmetry parameters (Nardelli, 1983) for the dihydro-isoxazole ring are q_2_ = 0.220 (2) Å, φ = 142.2 (4)° and Δ_s_(C_1_) = 1.1 (2)°, and for the piperidinone ring q_2_ = 0.074 (2) Å, q_3_ = 0.569 (2) Å, Q_T_ = 0.574 (2) Å and θ = 7.5 (2)°. The dihedral angle between the two benzene rings (C9—C14 and C16—C21) is 84.2 (1)°. The chlorine atom deviates by -0.065 (1) Å from the plane of the attached C16—C21 benzene ring, and the methyl carbon atom C15 deviates by 0.082 (2) Å from the plane of the C9—C14 benzene ring.

The crystal packing is stabilized by van der Waals forces.

### Experimental

To a well stirred mixture of 1-methyl-3-[(*E*)-4-methylphenylmethylidene]tetrahydro-4(1*H*)-pyridinone (1 mmol) and 4-chlorobenzohydroximoyl chloride (3 mmol) in benzene (15 ml), triethylamine (3 mmol) was added dropwise over a period of 10 min and stirring was continued for 5 h at ambient temperature. The triethylamine hydrochloride was filtered off, solvent evaporated *in vacuo*, and the product was purified by column chromatography using petroleum ether-ethyl acetate (4:1 *v*/*v*) mixture. The compound was then recrystallized from ethanol-ethyl acetate (1:1 *v*/*v*).

### Refinement

H atoms were positioned geometrically and allowed to ride on their parent atoms, with C—H = 0.93–0.98 Å and *U*_iso_(H) = 1.5*U*_eq_(methyl C) or 1.2*U*_eq_(C). The crystal used was an inversion twin.

### Crystal data

**Table:**

C_21_H_21_ClN_2_O_2_	*F*_000_ = 776
*M**_r_* = 368.85	*D*_x_ = 1.313 Mg m^−^^3^
Orthorhombic, *P*2_1_2_1_2_1_	Mo *K*α radiation λ = 0.71073 Å
Hall symbol: P 2ac 2ab	Cell parameters from 2169 reflections
*a* = 11.4585 (8) Å	θ = 2.2–25.0º
*b* = 16.1132 (11) Å	µ = 0.22 mm^−^^1^
*c* = 10.1038 (7) Å	*T* = 293 (2) K
*V* = 1865.5 (2) Å^3^	Block, colourless
*Z* = 4	0.24 × 0.23 × 0.20 mm

### Data collection

**Table:**

Bruker SMART APEX CCD area-detector diffractometer	3771 reflections with *I* > 2σ(*I*)
Radiation source: fine-focus sealed tube	*R*_int_ = 0.022
Monochromator: graphite	θ_max_ = 28.0º
*T* = 293(2) K	θ_min_ = 2.2º
ω scans	*h* = −14→14
Absorption correction: none	*k* = −20→20
16236 measured reflections	*l* = −12→13
4350 independent reflections	

### Refinement

**Table:**

Refinement on *F*^2^	Hydrogen site location: inferred from neighbouring sites
Least-squares matrix: full	H-atom parameters constrained
*R*[*F*^2^ > 2σ(*F*^2^)] = 0.043	*w* = 1/[σ^2^(*F*_o_^2^) + (0.0709*P*)^2^ + 0.0494*P*] where *P* = (*F*_o_^2^ + 2*F*_c_^2^)/3
*wR*(*F*^2^) = 0.111	(Δ/σ)_max_ = 0.001
*S* = 1.04	Δρ_max_ = 0.24 e Å^−^^3^
4350 reflections	Δρ_min_ = −0.16 e Å^−^^3^
238 parameters	Extinction correction: none
Primary atom site location: structure-invariant direct methods	Absolute structure: Flack (1983); 1846 Friedel pairs
Secondary atom site location: difference Fourier map	Flack parameter: 0.65 (6)

### Special details

**Table:**

Geometry. All e.s.d.'s (except the e.s.d. in the dihedral angle between two l.s. planes) are estimated using the full covariance matrix. The cell e.s.d.'s are taken into account individually in the estimation of e.s.d.'s in distances, angles and torsion angles; correlations between e.s.d.'s in cell parameters are only used when they are defined by crystal symmetry. An approximate (isotropic) treatment of cell e.s.d.'s is used for estimating e.s.d.'s involving l.s. planes.
Refinement. Refinement of *F*^2^ against ALL reflections. The weighted *R*-factor *wR* and goodness of fit *S* are based on *F*^2^, conventional *R*-factors *R* are based on *F*, with *F* set to zero for negative *F*^2^. The threshold expression of *F*^2^ > σ(*F*^2^) is used only for calculating *R*-factors(gt) *etc*. and is not relevant to the choice of reflections for refinement. *R*-factors based on *F*^2^ are statistically about twice as large as those based on *F*, and *R*- factors based on ALL data will be even larger.

### Fractional atomic coordinates and isotropic or equivalent isotropic displacement parameters (Å^2^)

**Table:**

	*x*	*y*	*z*	*U*_iso_*/*U*_eq_	
C1	0.39999 (14)	0.21740 (10)	0.26025 (16)	0.0441 (4)	
C2	0.47545 (14)	0.14014 (10)	0.28375 (16)	0.0432 (4)	
H2	0.5027	0.1400	0.3757	0.052*	
C3	0.57576 (15)	0.16160 (10)	0.19396 (17)	0.0451 (4)	
C4	0.26874 (15)	0.20376 (11)	0.2506 (2)	0.0498 (4)	
H4A	0.2523	0.1668	0.1771	0.060*	
H4B	0.2414	0.1772	0.3310	0.060*	
C5	0.2264 (2)	0.33753 (12)	0.3408 (2)	0.0643 (6)	
H5A	0.2049	0.3109	0.4233	0.077*	
H5B	0.1784	0.3866	0.3301	0.077*	
C6	0.3552 (2)	0.36245 (12)	0.3452 (3)	0.0666 (6)	
H6A	0.3773	0.3897	0.2633	0.080*	
H6B	0.3691	0.4005	0.4179	0.080*	
C7	0.42530 (17)	0.28489 (11)	0.36367 (19)	0.0516 (4)	
C8	0.08128 (17)	0.26456 (16)	0.2160 (2)	0.0703 (6)	
H8A	0.0523	0.2374	0.2939	0.106*	
H8B	0.0692	0.2295	0.1405	0.106*	
H8C	0.0404	0.3160	0.2035	0.106*	
C9	0.42089 (13)	0.05732 (10)	0.25237 (16)	0.0422 (3)	
C10	0.40319 (16)	0.03083 (11)	0.12336 (18)	0.0510 (4)	
H10	0.4296	0.0634	0.0535	0.061*	
C11	0.34703 (17)	−0.04313 (12)	0.0970 (2)	0.0567 (5)	
H11	0.3365	−0.0596	0.0096	0.068*	
C12	0.30606 (15)	−0.09324 (11)	0.1981 (2)	0.0535 (4)	
C13	0.32650 (18)	−0.06790 (12)	0.3258 (2)	0.0590 (5)	
H13	0.3018	−0.1012	0.3955	0.071*	
C14	0.38319 (16)	0.00627 (11)	0.35339 (18)	0.0520 (4)	
H14	0.3959	0.0217	0.4409	0.062*	
C15	0.2395 (2)	−0.17151 (13)	0.1691 (3)	0.0755 (7)	
H15A	0.2165	−0.1972	0.2507	0.113*	
H15B	0.2882	−0.2090	0.1199	0.113*	
H15C	0.1713	−0.1584	0.1180	0.113*	
C16	0.69030 (15)	0.12007 (10)	0.19526 (18)	0.0453 (4)	
C17	0.77102 (16)	0.13329 (12)	0.09496 (19)	0.0522 (4)	
H17	0.7506	0.1654	0.0221	0.063*	
C18	0.88106 (17)	0.09925 (12)	0.1024 (2)	0.0559 (4)	
H18	0.9347	0.1079	0.0347	0.067*	
C19	0.91083 (15)	0.05239 (11)	0.2108 (2)	0.0526 (4)	
C20	0.83202 (17)	0.03571 (11)	0.30978 (19)	0.0559 (5)	
H20	0.8526	0.0022	0.3810	0.067*	
C21	0.72139 (17)	0.06986 (12)	0.30132 (19)	0.0527 (4)	
H21	0.6671	0.0590	0.3676	0.063*	
N1	0.55545 (14)	0.22078 (9)	0.11384 (15)	0.0530 (4)	
N2	0.20607 (14)	0.28110 (9)	0.23159 (16)	0.0548 (4)	
O1	0.44115 (11)	0.25059 (8)	0.13460 (12)	0.0552 (3)	
O2	0.49219 (14)	0.27449 (9)	0.45270 (15)	0.0714 (4)	
Cl1	1.05097 (4)	0.01181 (4)	0.22360 (6)	0.07411 (18)	

### Atomic displacement parameters (Å^2^)

**Table:**

	*U*^11^	*U*^22^	*U*^33^	*U*^12^	*U*^13^	*U*^23^
C1	0.0457 (8)	0.0424 (8)	0.0441 (9)	0.0017 (7)	0.0011 (7)	0.0028 (6)
C2	0.0438 (8)	0.0449 (8)	0.0410 (8)	0.0035 (6)	−0.0025 (7)	0.0023 (7)
C3	0.0428 (8)	0.0451 (8)	0.0476 (9)	−0.0007 (7)	−0.0009 (7)	−0.0016 (7)
C4	0.0457 (8)	0.0503 (9)	0.0532 (10)	0.0026 (7)	−0.0022 (8)	0.0016 (8)
C5	0.0689 (13)	0.0495 (10)	0.0744 (14)	0.0179 (9)	0.0117 (11)	0.0037 (9)
C6	0.0768 (14)	0.0425 (9)	0.0805 (14)	0.0026 (9)	0.0013 (12)	−0.0085 (9)
C7	0.0498 (10)	0.0469 (9)	0.0580 (10)	−0.0047 (7)	0.0042 (9)	0.0014 (8)
C8	0.0503 (10)	0.0856 (14)	0.0751 (13)	0.0143 (10)	0.0057 (10)	0.0159 (12)
C9	0.0379 (7)	0.0420 (8)	0.0467 (9)	0.0046 (6)	0.0004 (7)	0.0029 (6)
C10	0.0549 (10)	0.0498 (9)	0.0482 (9)	0.0000 (8)	0.0031 (8)	0.0028 (7)
C11	0.0554 (10)	0.0553 (11)	0.0595 (11)	0.0033 (8)	−0.0044 (9)	−0.0067 (9)
C12	0.0407 (8)	0.0428 (9)	0.0769 (13)	0.0073 (7)	−0.0018 (9)	−0.0003 (8)
C13	0.0582 (11)	0.0477 (9)	0.0710 (13)	0.0022 (9)	0.0080 (9)	0.0151 (9)
C14	0.0563 (10)	0.0509 (9)	0.0489 (9)	0.0031 (8)	0.0013 (8)	0.0037 (8)
C15	0.0590 (12)	0.0509 (11)	0.116 (2)	−0.0032 (9)	−0.0063 (13)	−0.0041 (12)
C16	0.0425 (8)	0.0422 (8)	0.0510 (9)	−0.0008 (7)	−0.0021 (7)	−0.0060 (7)
C17	0.0485 (9)	0.0548 (10)	0.0534 (10)	0.0006 (8)	0.0014 (8)	0.0024 (8)
C18	0.0465 (9)	0.0619 (11)	0.0594 (11)	0.0015 (8)	0.0070 (9)	−0.0020 (9)
C19	0.0428 (8)	0.0517 (9)	0.0634 (11)	0.0056 (7)	−0.0035 (8)	−0.0114 (8)
C20	0.0586 (11)	0.0551 (10)	0.0541 (10)	0.0111 (8)	−0.0027 (9)	0.0004 (8)
C21	0.0505 (9)	0.0547 (10)	0.0527 (10)	0.0035 (8)	0.0059 (8)	0.0000 (8)
N1	0.0471 (8)	0.0580 (9)	0.0540 (8)	0.0063 (7)	0.0061 (7)	0.0053 (7)
N2	0.0495 (8)	0.0569 (9)	0.0580 (9)	0.0087 (7)	0.0036 (7)	0.0104 (7)
O1	0.0519 (7)	0.0619 (7)	0.0519 (6)	0.0132 (6)	0.0056 (6)	0.0146 (6)
O2	0.0826 (11)	0.0639 (9)	0.0678 (9)	−0.0015 (8)	−0.0190 (8)	−0.0105 (7)
Cl1	0.0503 (3)	0.0879 (4)	0.0841 (4)	0.0210 (2)	−0.0020 (2)	−0.0061 (3)

### Geometric parameters (Å, °)

**Table:**

C1—O1	1.456 (2)	C9—C10	1.386 (2)
C1—C4	1.523 (2)	C10—C11	1.380 (3)
C1—C2	1.534 (2)	C10—H10	0.93
C1—C7	1.536 (2)	C11—C12	1.384 (3)
C2—C3	1.505 (2)	C11—H11	0.93
C2—C9	1.507 (2)	C12—C13	1.374 (3)
C2—H2	0.98	C12—C15	1.503 (3)
C3—N1	1.272 (2)	C13—C14	1.389 (3)
C3—C16	1.473 (2)	C13—H13	0.93
C4—N2	1.451 (2)	C14—H14	0.93
C4—H4A	0.97	C15—H15A	0.96
C4—H4B	0.97	C15—H15B	0.96
C5—N2	1.448 (3)	C15—H15C	0.96
C5—C6	1.530 (3)	C16—C17	1.388 (3)
C5—H5A	0.97	C16—C21	1.389 (3)
C5—H5B	0.97	C17—C18	1.377 (3)
C6—C7	1.497 (3)	C17—H17	0.93
C6—H6A	0.97	C18—C19	1.373 (3)
C6—H6B	0.97	C18—H18	0.93
C7—O2	1.194 (2)	C19—C20	1.374 (3)
C8—N2	1.463 (3)	C19—Cl1	1.7386 (17)
C8—H8A	0.96	C20—C21	1.385 (3)
C8—H8B	0.96	C20—H20	0.93
C8—H8C	0.96	C21—H21	0.93
C9—C14	1.380 (2)	N1—O1	1.411 (2)
			
O1—C1—C4	108.48 (14)	C11—C10—C9	121.03 (17)
O1—C1—C2	104.50 (13)	C11—C10—H10	119.5
C4—C1—C2	116.71 (14)	C9—C10—H10	119.5
O1—C1—C7	105.78 (13)	C10—C11—C12	121.29 (19)
C4—C1—C7	109.40 (14)	C10—C11—H11	119.4
C2—C1—C7	111.27 (14)	C12—C11—H11	119.4
C3—C2—C9	113.17 (14)	C13—C12—C11	117.52 (17)
C3—C2—C1	98.68 (13)	C13—C12—C15	121.3 (2)
C9—C2—C1	116.88 (13)	C11—C12—C15	121.2 (2)
C3—C2—H2	109.2	C12—C13—C14	121.60 (18)
C9—C2—H2	109.2	C12—C13—H13	119.2
C1—C2—H2	109.2	C14—C13—H13	119.2
N1—C3—C16	120.60 (16)	C9—C14—C13	120.73 (17)
N1—C3—C2	114.59 (15)	C9—C14—H14	119.6
C16—C3—C2	124.81 (14)	C13—C14—H14	119.6
N2—C4—C1	111.92 (15)	C12—C15—H15A	109.5
N2—C4—H4A	109.2	C12—C15—H15B	109.5
C1—C4—H4A	109.2	H15A—C15—H15B	109.5
N2—C4—H4B	109.2	C12—C15—H15C	109.5
C1—C4—H4B	109.2	H15A—C15—H15C	109.5
H4A—C4—H4B	107.9	H15B—C15—H15C	109.5
N2—C5—C6	110.03 (17)	C17—C16—C21	118.79 (16)
N2—C5—H5A	109.7	C17—C16—C3	121.15 (16)
C6—C5—H5A	109.7	C21—C16—C3	119.99 (16)
N2—C5—H5B	109.7	C18—C17—C16	120.60 (18)
C6—C5—H5B	109.7	C18—C17—H17	119.7
H5A—C5—H5B	108.2	C16—C17—H17	119.7
C7—C6—C5	107.56 (16)	C19—C18—C17	119.33 (18)
C7—C6—H6A	110.2	C19—C18—H18	120.3
C5—C6—H6A	110.2	C17—C18—H18	120.3
C7—C6—H6B	110.2	C18—C19—C20	121.66 (16)
C5—C6—H6B	110.2	C18—C19—Cl1	119.72 (15)
H6A—C6—H6B	108.5	C20—C19—Cl1	118.62 (15)
O2—C7—C6	123.75 (19)	C19—C20—C21	118.62 (17)
O2—C7—C1	122.32 (16)	C19—C20—H20	120.7
C6—C7—C1	113.88 (17)	C21—C20—H20	120.7
N2—C8—H8A	109.5	C20—C21—C16	120.92 (17)
N2—C8—H8B	109.5	C20—C21—H21	119.5
H8A—C8—H8B	109.5	C16—C21—H21	119.5
N2—C8—H8C	109.5	C3—N1—O1	109.30 (14)
H8A—C8—H8C	109.5	C5—N2—C4	111.01 (15)
H8B—C8—H8C	109.5	C5—N2—C8	110.72 (16)
C14—C9—C10	117.78 (16)	C4—N2—C8	109.97 (16)
C14—C9—C2	120.12 (15)	N1—O1—C1	107.77 (12)
C10—C9—C2	122.07 (15)		
			
O1—C1—C2—C3	20.69 (15)	C10—C11—C12—C15	176.79 (18)
C4—C1—C2—C3	140.47 (15)	C11—C12—C13—C14	1.8 (3)
C7—C1—C2—C3	−93.02 (15)	C15—C12—C13—C14	−176.97 (18)
O1—C1—C2—C9	−100.88 (16)	C10—C9—C14—C13	−1.8 (3)
C4—C1—C2—C9	18.9 (2)	C2—C9—C14—C13	176.12 (16)
C7—C1—C2—C9	145.41 (15)	C12—C13—C14—C9	0.1 (3)
C9—C2—C3—N1	110.31 (17)	N1—C3—C16—C17	−11.9 (3)
C1—C2—C3—N1	−13.93 (19)	C2—C3—C16—C17	168.66 (16)
C9—C2—C3—C16	−70.2 (2)	N1—C3—C16—C21	164.90 (17)
C1—C2—C3—C16	165.53 (15)	C2—C3—C16—C21	−14.5 (3)
O1—C1—C4—N2	−63.77 (19)	C21—C16—C17—C18	−1.9 (3)
C2—C1—C4—N2	178.61 (15)	C3—C16—C17—C18	174.95 (17)
C7—C1—C4—N2	51.2 (2)	C16—C17—C18—C19	−0.5 (3)
N2—C5—C6—C7	−60.1 (2)	C17—C18—C19—C20	2.7 (3)
C5—C6—C7—O2	−123.4 (2)	C17—C18—C19—Cl1	−178.00 (15)
C5—C6—C7—C1	54.2 (2)	C18—C19—C20—C21	−2.4 (3)
O1—C1—C7—O2	−115.9 (2)	Cl1—C19—C20—C21	178.29 (14)
C4—C1—C7—O2	127.43 (19)	C19—C20—C21—C16	−0.1 (3)
C2—C1—C7—O2	−3.0 (2)	C17—C16—C21—C20	2.2 (3)
O1—C1—C7—C6	66.42 (19)	C3—C16—C21—C20	−174.70 (17)
C4—C1—C7—C6	−50.2 (2)	C16—C3—N1—O1	−178.78 (14)
C2—C1—C7—C6	179.33 (16)	C2—C3—N1—O1	0.7 (2)
C3—C2—C9—C14	141.75 (16)	C6—C5—N2—C4	64.4 (2)
C1—C2—C9—C14	−104.61 (18)	C6—C5—N2—C8	−173.13 (17)
C3—C2—C9—C10	−40.4 (2)	C1—C4—N2—C5	−60.1 (2)
C1—C2—C9—C10	73.2 (2)	C1—C4—N2—C8	177.08 (16)
C14—C9—C10—C11	1.6 (3)	C3—N1—O1—C1	14.11 (18)
C2—C9—C10—C11	−176.26 (16)	C4—C1—O1—N1	−147.46 (14)
C9—C10—C11—C12	0.3 (3)	C2—C1—O1—N1	−22.29 (16)
C10—C11—C12—C13	−2.0 (3)	C7—C1—O1—N1	95.25 (15)
